# A novel strategy facilitating endoscopic submucosal dissection of proximal colonic lesions: the rubber band and sheath method

**DOI:** 10.1055/a-2063-3291

**Published:** 2023-04-26

**Authors:** Hirofumi Abe, Takashi Toyonaga, Douglas Motomura, Ryosuke Ishida, Hiroya Sakaguchi, Tetsuya Yoshizaki, Yuzo Kodama

**Affiliations:** 1 Division of Gastroenterology, Department of Internal Medicine, Kobe University Graduate School of Medicine, Kobe, Japan; 2 Department of Endoscopy, Kobe University Hospital, Kobe, Japan; 3 Department of Gastroenterology, University of British Columbia, Vancouver, British Columbia, Canada

A 70-year-old woman was referred to our hospital for the treatment of a laterally spreading tumor in the ascending colon ( Fig. 1 ). Endoscopic submucosal dissection (ESD) was performed using a novel strategy with an orthodontic elastic rubber band (Latex Elastics Light 8 mm; Tomy International, Osaka, Japan) and EndoTrac (EndoTrac T type; Top, Tokyo, Japan) ( Fig. 2 , Video 1 ) 1 . The latter is a traction device composed of a plastic sheath and a line with an adjustable loop at its tip.

**Fig. 1:**
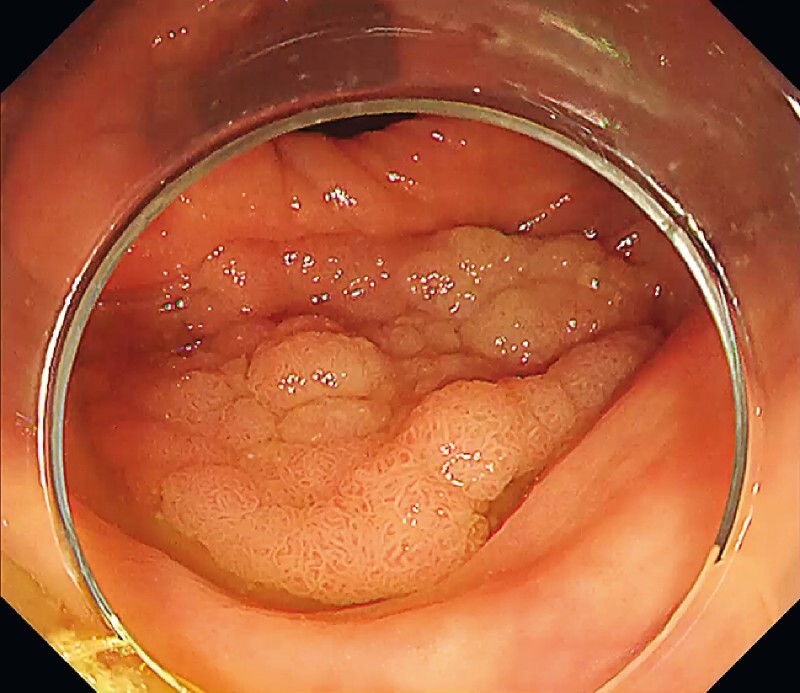
White-light view of the laterally spreading tumor in the ascending colon.

**Fig. 2:**
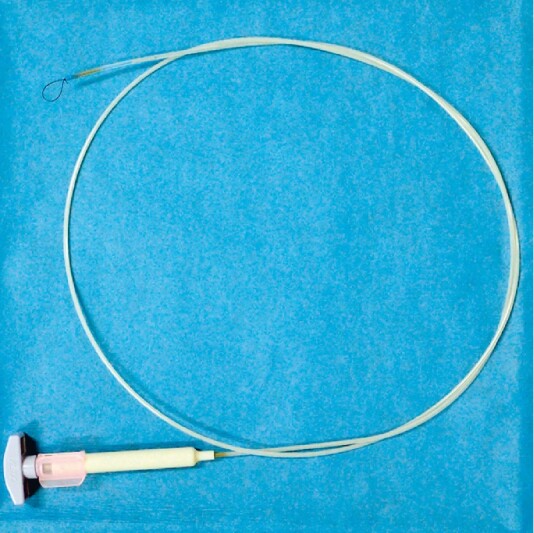
The EndoTrac T type (Top, Tokyo, Japan), which is a traction device, is composed of a plastic sheath and a line with an adjustable loop at its tip.

**Video 1** The rubber band and sheath method for endoscopic submucosal dissection of proximal colonic lesions.


For preparation, the rubber band was tied to the tip of EndoTrac and wrapped around the endoscope hood twice. Both the endoscope and EndoTrac were inserted simultaneously.

ESD was started from the partial mucosal incision at the anal side of the lesion, followed by deeper cutting. Then, the EndoTrac was released by pulling on the sheath while the endoscope was in the retroflexed position ( Fig. 3 a ). The rubber band was grasped by a rotatable clip, which could be reopened multiple times (SureClip mini; Micro-Tech, Nanjing, China). The clip was anchored to the mucosal incision site. Adjustment of traction was possible by pushing or pulling the sheath while changing the length of the string. Pushing the sheath to the oral side was useful for exposing the submucosal layer, resulting in easy creation of the mucosal flap ( Fig. 3 b ). Once the flap was created, the circumferential incision was completed and submucosal dissection was started. Pulling the sheath to the anal side was helpful for maintaining traction during this process ( Fig. 3 c ). En bloc resection was uneventfully accomplished.

**Fig. 3:**
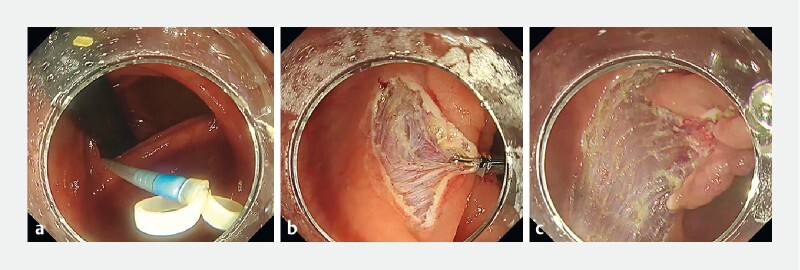
Use of the EndoTrac device (Top, Tokyo, Japan) during endoscopic submucosal dissection. **a** The device was released by pulling the sheath while the endoscope was in a retroflexed position. **b** Pushing the sheath to the oral side was useful for exposing the submucosal layer. **c** Pulling the sheath to the anal side was helpful for maintaining the traction.

Traction devices are useful in ESD, and manipulating the direction of tension during ESD is often desired to overcome challenging situations. Although this method may be challenging if insertion of the colonoscope is difficult and the lumen is too narrow for the retroflexed position, it enables us to deliver adjustable traction without endoscope reinsertion, and facilitates ESD for difficult lesions in the proximal colon.

Endoscopy_UCTN_Code_TTT_1AQ_2AD

## Notes

Endoscopy E-Videos

https://eref.thieme.de/e-videos
*E-Videos* is an open access online section of the journal *Endoscopy* , reporting on interesting cases and new techniques in gastroenterological endoscopy. All papers include a high-quality video and are published with a Creative Commons CC-BY license. Endoscopy E-Videos qualify for HINARI discounts and waivers and eligibility is automatically checked during the submission process. We grant 100% waivers to articles whose corresponding authors are based in Group A countries and 50% waivers to those who are based in Group B countries as classified by Research4Life (see: https://www.research4life.org/access/eligibility/ ). This section has its own submission website at https://mc.manuscriptcentral.com/e-videos
